# The Role of Phonological Factors in the Processing of Polish Phonotactics

**DOI:** 10.1177/00238309251327671

**Published:** 2025-08-29

**Authors:** Paula Orzechowska, Andrzej Porębski, Marta Nowak

**Affiliations:** Faculty of English, Adam Mickiewicz University in Poznań, Poland; Faculty of Law and Administration, Jagiellonian University, Kraków, Poland; Adam Mickiewicz University in Poznań, Poland

**Keywords:** Phonotactics, sonority, vowel quality, reaction times, Polish

## Abstract

One of the predominant questions asked in phonological research refers to the way in which strings of vowels and consonants are perceived and processed by native speakers. In this paper, we make an attempt at uncovering the mental processes that underlie the online processing of phonotactics in Polish; a language featuring an unusual array of strings of consonants. We report on a reaction time experiment using nonce monosyllables with final consonant clusters and identify phonological factors that determine their acceptability. The factors include cluster (non-)existence in the lexicon of Polish, cluster well-formedness in terms of the universally preferred sonority slope, and the quality of the nuclear vowel. The findings testify to the facilitative role of cluster existence and well-formedness on phonotactic intuitions. That is, universally preferred and existent clusters are easily identified as possible and involve the shortest reaction times. Moreover, we detected a systematic perceptual contribution of vowels, whereby the front-back dimension (rather than sonority-related high-low dimension) seems to facilitate the decision-making process.

## 1 Introduction

Constraints on admissible sequences of segments in a language constitute one of the core areas of phonological research. For over a century, attention has been drawn to the restrictions that regulate the structure of a universally preferred syllable. Such restriction refers to the number of consonants admissible at syllable margins (e.g., Gordon, 2016; Greenberg, 1978), the principles of their syntagmatic organization based on, for instance, the minimal phonological contrast (Trnka, 1939), sound loudness (or sonority, Jespersen, 1904; Parker, 2002; Selkirk, 1984) or the (non-)identical place of articulation (Leben, 1973; McCarthy, 1988) (see Parker, 2017 and numerous references therein). Although focus has been placed on restrictions between sequences of consonants (or clusters), some studies have investigated the possible consonant-vowel combinations (e.g., Kessler & Treiman, 1997; Randolph, 1989; Trnka, 1966). With the advent of psycholinguistic methods, researchers have investigated cognitive mechanisms that underlie the processing of phonological properties of adjacent segments (e.g., Berent et al., 2007; Moreton, 2002; Pitt, 1998).

The present paper casts light on the cognitive representations of sub-lexical properties of Polish, a language that displays “enormous complexity of consonantal sequences” (Rochoń, 2000, p. 1) and numerous “odd-looking” (Cyran & Gussmann, 1999, p. 219f.) and “unusual” (Rubach & Booij, 1990, p. 454) clusters. To reach this goal, we investigate native speakers’ intuitions concerning the phonological properties of vowels and two-member clusters following them.

## 2 Phonotactics and phonotactic principles

Complex consonant clusters in Polish are attributed to the violations of phonological principles and universals, the lack of syllabic consonants, and the intervention of morphology. Moreover, such complex structures are by no means rare. The uniqueness of Polish phonotactics is reflected in the fact that all these complexity aspects can co-occur in a single cluster type, as illustrated in (1).

(1) Examples of complex clusters in Polish
   (1a) initial:   /fkw/ *w* *+* *kładać* ‘to put into’-perfective
          /brvj/ *brwiowy* ‘eyebrow-like’
          /drgn/ *drg* *+* *nąć* ‘to vibrate’-perfective
   (1b) final:   /jɕʨ/ *przyjś* *+* *ć* ‘to come’
          /wdw/ *zwięd* *+* *ł* ‘(a flower) wilted’
          /mpstf/ *przestępstw* *+* ∅ ‘crime’-genitive plural

Consonant sequences in (1a) and (1b) violate the universal syllable structure, which is typically represented as a combination of a consonant (C) and a vowel (V) (the so-called CV, e.g., Greenberg, 1978; Maddieson, 2013), and—with the exception of /jɕʨ/—are phonologically marked. Here, the markedness status of a cluster is determined by the *Sonority Sequencing Generalization*, which we shall refer to as the sonority principle (Jespersen, 1904; Selkirk, 1984; Steriade, 1982). It requires that the sonority level of segments increase from the syllable margins toward the nucleus. Following the sonority hierarchy of Goldsmith (1990) (vowel > glide > liquid > nasal > fricative > affricate > plosive), in a well-formed monosyllable *cramp* /kræmp/, /r m/ adjacent to the vowel displays higher sonority than /k p/ found at the syllable margins, which is the case with most of onset and coda clusters in English (Trnka, 1966). In Polish, however, violations of the principle are relatively common. One of the reasons is that complex clusters can be triggered by morphology (Dressler & Dziubalska-Kołaczyk, 2006), for instance, in the affixation of perfectivizing morphemes {w-, -nąć}, which generate initial clusters in *w* *+* *kładać* and *drg* *+* *nąć in* (1a) or in the truncation of {-o} in genitive plural forms, which generates the longest final five-member cluster in *przestępstw* (← *przestępstw* *+* *o*-nominative singular) (1b).

Such long and sonority-violating clusters have been well-documented, for example, in theoretical accounts on the syllable (e.g., Cyran & Gussmann, 1999; Rochoń, 2000; Rubach & Booij, 1990), or in corpus studies on word edge phonotactics (e.g., Dobrogowska, 1992; Orzechowska, 2019; Zydorowicz et al., 2016). As to word margins, the sources have listed over 400 cluster types in word initial position and 200 types in word-final position, a third of which is phonotactically marked. For example, Orzechowska (2019) reported that out of 759 cluster types permitted at word margins, 351 (= 46%) violate the sonority principle.

The sonority hierarchy has been also established for vowels. It has been viewed as a function of height and peripherality. Vowel peripherality in the acoustic space teases apart the central vowels from the others, while height further specifies the degree of lowering of the mandible. It has been generally agreed that higher vowels are less sonorous than lower vowels (e.g., Dell & Elmedlaoui, 1985; Goldsmith, 1990; Parker, 2002), and that the latter are preferred as syllable nuclei (e.g., Gordon et al., 2012; Kenstowicz, 1996). Given these sonority hierarchies and the distribution of six Polish vowels in the vowel space (Jassem, 2003), the following sonority hierarchy can be proposed: low /a ɛ ɔ/ > high /i u/ > high central /ɨ/.

From the aforementioned restrictions, we can derive a VCC structure that best serves perceptibility and facilitates processing: The nucleus should contain vowel /a/, and the final cluster should display a decrease in sonority. Following a common view, which relates the sonority hierarchy to the “inherent loudness of individual segment-types” (Laver, 1994, p. 156), resulting in well-defined acoustic correlates (e.g., Blevins, 1995; Gordon et al., 2012; Parker, 2002), sequences composed of /a/ + sonority fall are expected to be universally preferred in production, acoustically more salient than sequences violating the sonority profile, and in consequence, facilitate perception and minimize cognitive load. The facilitating and hindering effects of well-formed and ill-formed syllables for perception are outlined in Section 3.

## 3 Processing of phonotactic constraints (in Polish)

A reliable source of information about the implicit knowledge of the phonotactic grammar of a language comes from the way in which native speakers apply this knowledge to novel words. Thus, in psycho- and neuro-linguistic research, the nonce-word paradigm has been employed to investigate the principles underlying the internal organization of words in terms of legal and illegal phonotactics (e.g., Domahs et al., 2009; Rossi et al., 2011; Wiese et al., 2017), the hierarchical organization of the syllable (e.g., Treiman, 1984; Treiman et al., 1995), and phoneme probabilities (e.g., Vitevitch & Luce, 1999; Weber & Cutler, 2006). Generally, it has been demonstrated that novel sequences of sounds tend to be misperceived and mispronounced and filtered through the phonotactic possibilities of the mother tongue. That is, illicit phonotactics entails a strong perceptual bias toward a familiar form (e.g., Dupoux et al., 1999; Hallé et al., 1998) and different articulatory coordination compared to forms existent in the native language (e.g., Browman & Goldstein, 1992; Davidson, 2006). Thus, in phonological research, novel items and illicit phonotactics have been used to investigate the organization of grammar in the minds of speakers.

An example of such an illegal initial cluster in English is /tl/. Although the cluster is common across the languages of the world (Greenberg, 1978), represents the most natural ordering of consonants (Clements, 1990), and is difficult neither for production nor perception (Blevins & Grawunder, 2009), it is disfavoured by native speakers of English and is systematically rejected in experimental conditions. Pitt (1998) showed that speakers misperceive /tlV/ as disyllabic /tela/, while the effect was not observed for existent syllable onsets. Hallé and Best (2007) demonstrated that listeners tend to categorize the initial plosives in /tl dl/ as /k g/, respectively, suggesting that non-existent clusters undergo phonotactic perceptual assimilation to the phonetically most similar existing clusters. Similar observations were made for other sonority-obeying and unattested cluster types. Moreton’s (2002) work on nonce onsets /dl bw/ showed a perceptual bias only against /dl/ due to smaller sonority distance. The findings were confirmed in an fMRI study in Berent et al. (2014) who demonstrated that the hemodynamic response in the brain is modulated by sonority distances in onsets, with larger distances being preferred over small distances (i.e., large rise in /blif/ > small rise in /bnif/ > plateau in /bdif/ > fall in /lbif/). Daland et al. (2011) argued that onset clusters representing more severe phonotactic violations (i.e., sonority fall) were evaluated as worse than clusters with no sonority differential (i.e., plateaus) in English. Similar questions on the processing of (il)licit, familiar and sonority-violating phonotactics were asked in a series of online and offline studies on Polish (Orzechowska, 2019; Szpyra-Kozłowska & Zydorowicz, 2020; Wagner et al., 2012; Wiese et al., 2017).

The nonce-word paradigm is employed to determine the cognitive resources allocated to completing different tasks. For example, Wagner et al. (2012) investigated the perception of /pt/ by native English and Polish speakers and concluded that in onset position, the cluster is correctly identified only by the latter group of subjects. Polish speakers distinguish between nonce words starting with an existent cluster /pt/ (e.g., *ptak* ‘bird’) and with a syllable /pet/ (e.g., *petycja* ‘petition’), in contrast to English speakers, who misperceive /pt/ despite the presence of the /pt/ vs. /pet/ contrast word-finally (*kept* vs. *trumpet*). The findings suggest that the exposure to prosodically-constrained phoneme sequences determines accurate perception.

In a learnability study using event-related potentials (henceforth ERPs), Wiese et al. (2017) asked subjects to learn nonce monosyllables containing final well-formed and ill-formed CCs, for example, /fars/, /nɔpx/. The study compared brain responses to three conditions: cluster existence (existent vs. non-existent), its sonority slope (well-formed vs. ill-formed), and learning spread across several days (EEG 1 vs. EEG 2). The facilitating effect of existent and WF clusters (e.g., /jp lk rs/) for processing was identified only in the late time window (700–1,050 ms), which is generally associated with learning and attention, and not in the early time window (450–550 ms), associated with the processing of nonce words, pseudowords or neologisms. As regards the behavioral data, the main effects were reported for exposure: correctness rates increased from the first to the second EEG session.

It can be thus speculated that other phonological factors play a role in accepting existent and non-existent clusters in Polish. To test this hypothesis, Orzechowska (2019) measured reaction times of native speakers to the same set of stimuli as in Wiese et al. (2017). In addition, the clusters were matched in terms of the place of articulation distance between consonants, for example, /fars/ (well-formed, dist = 1), /nɔpx/ (ill-formed, dist = 6). The results testified to a division of labor between the manner of articulation and the place of articulation features. Although accuracy rates were the highest for well-formed existent CCs, processing was facilitated by large place distances. The shortest latencies were observed for sequences of labial and dorsal consonants in either order (e.g., /mk gb xm/), compared to, for instance, dental-dental, alveolar-palatoalveolar sequences, regardless of their sonority profile and existence. The findings point to a lower cognitive load in the processing of place features and suggest that perceived place constraints outweigh perceived manner constraints.

Polish speakers’ sensitivity to sonority violations on the metalinguistic level was also reported in an offline study in Szpyra-Kozłowska and Zydorowicz (2020), which measured acceptability responses to different CCs embedded in nonce words. The study suggested that the majority of accepted items (75% of words with initial CCs; 61.5% of words with final CCs) follow the sonority principle. This result aligns with Orzechowska (2019), who reported the facilitating effect of sonority in the accuracy data.

## 4 The study

### 4.1 Goals

The goal of the study is to test the contribution of three word-internal factors to the processing of novel forms: cluster existence, its phonological structure in terms of sonority, and the phonological properties of the neighboring vowel. Another question is whether the findings reported in previous studies on the processing of Polish final phonotactics (e.g., Orzechowska, 2019; Wiese et al., 2017) can be corroborated when new variables are considered (here vowel quality). Eventually, the statistical analyses have the potential of revealing a (relative) functional load of phonological primitives such as vowel features and principles of segmental organization.

### 4.2 Conditions

This study tests three phonotactic conditions: (1) the existence of a cluster, (2) its sonority profile, and (3) the quality of the preceding vowel.

The existence condition involves two levels: existent clusters (EX), which are attested in Polish word-finally, and non-existent clusters (NEX), which are absent in word-final position (based on corpus resources, see Section 2).

The sonority condition specifies the sonority profile of clusters based on the scale of Goldsmith (1990). We introduce a three-fold division of final clusters into well-formed (WF), which are characterized by a sonority fall; ill-formed (IF), which display a sonority rise; and plateau clusters. The latter class encompasses clusters with same sonority level. The inclusion of this category is motivated by its dubious status in phonological theory. Plateaus have been deemed universally disfavoured (Parker, 2002); however, this effect can be overridden by some language-specific constraints (e.g., Rubach & Booij, 1990). The question is whether plateaus are evaluated and processed more similarly to sonority-obeying or sonority-offending sequences.

The vowel quality condition involves three levels: /ɛ/, /a/, and /ɔ/. Out of six vowels available, the selection of /e a o/ allows for an evenly distributed representation of the vowels in the low dimension of the vowel chart (Jassem, 2003). The inclusion of these qualities can reveal whether Polish speakers are sensitive to vowel frontness (front /ɛ/ vs. central /a/ vs. back /ɔ/) or height (half-open /ɛ ɔ/ vs. open /a/).^1^

### 4.3 Hypotheses

We expect to observe an effect of existence and sonority on the response variable (i.e., accepting an item as a possible word) and reaction times (i.e., time needed to provide an answer measured in ms). Given the nature of the experimental task, our predictions on existence and the sonority slope are based on the previous findings for Polish (see Section 3.1.).

Specific hypotheses (H) for behavioral data (response) are as follows:

(H1) existence is *significant*: subjects are more likely to give a “yes” response to words containing existent clusters than to words with non-existent clusters.
(H2) sonority is *significant*: subjects are more likely to give a “yes” response to words containing well-formed clusters than to words with ill-formed clusters.
(H3) To be further inferred from (H1) and (H2): the highest rate of CVCC monosyllables accepted as potential Polish words is expected in existent and sonority-obeying forms.

A specific hypothesis for processing data (reaction times) is the following:

(H4) existence and sonority (and their interaction) are *non-significant*: the time needed to make a decision is neither affected by clusters’ existence status nor their sonority profile. It is likely that other factors affect response latencies.

As the effects of vowel quality on the processing of syllables have not been reported in the literature on Polish (compare with a similar study on German, Orzechowska & Wiese, 2024), there are at least two potential options (O) regarding the contribution of vowels:

(O1) vowel quality is *non-significant*: suggests that vowel quality is not consulted when processing consonant phonotactics,
(O2) vowel quality is *significant*: suggests that vowel quality facilitates phonological access.

The decision-making process on the acceptability of a form is related to cognitive effort (for an overview see Westbrook & Braver, 2015 and references therein). In its broadest sense, the term has been coupled with attention and difficulty: the execution of high-engagement tasks requires the allocation of cognitive resources, extra attention, and working memory. In line with the previous studies, we assume that longer reaction times reflect greater difficulty, cognitive cost, and more attention needed to process a word.

### 4.4 Subjects

Forty-one native speakers of Polish volunteered to take part in the study. The subjects (mean age = 22; 8 males, 33 females) were students at Adam Mickiewicz University (henceforth AMU) in Poznań. The largest number of students was recruited from the Faculty of English. Most of the subjects came from the Great Poland region. In the questionnaire completed prior to the experiment, no subject reported having visual or hearing disorders, or being under the influence of medication affecting reaction times and attention. The majority of subjects were right-handed (93%).

### 4.5 Stimuli and apparatus

Stimuli were nonce monosyllables CVCC. In the selection of WF-IF pairs, we first included clusters for which equivalents with the reversed ordering of segments were available, for instance, WF /-sp/ versus IF /-ps/, or with the same voicing characteristics, for example, WF /-rk/ versus /-tr/. WF and IF clusters were also matched in terms of their logarithmic token frequencies (base 10) extracted from the corpus of the *Rzeczpospolita* newspaper (2000–2001; see Zydorowicz et al., 2016 for a detailed presentation). The log frequencies, spanned on a 6-point scale,^2^ were used to match /-sp/ and /-ps/ (log freq = 3), as well as /-rk/ (log freq = 3) and /-tr/ (log freq = 2). The final set embraced 33 existent cluster types: 15 WF, 15 IF, and 3 plateaus. The corresponding set of 33 NEX types was constructed in a way to ensure maximum phonetic similarity to the existent clusters.^3^ The complete list of cluster types is given in Table 1 and Appendix A.

**Table 1.** Clusters Used as Stimuli, Ordered Alphabetically.

	Existent	Non-existent
Plateau	mn, pt, ʃx	nm, tk, fs
Well-formed	fʦ, ft, js, lk, ŋk, rk, rɲ, rʦ, sk, sp, ɕʨ, wk, wp, wt, wʧ	fp, jb, jd, jg, jz, lg, ln, mg, rʤ, rg, sʦ, ʃk, wʣ, xp, xʨ
Ill-formed	fn, kl, kw, kx, nr, pl, pɲ, ps, pw, sm, sw, tf, tm, tr, tw	bn, bw, dw, ʣl, ʣw, ʥr, gl, gn, gw, mr, pf, px, tx, ʦf, ʨs

To form a monosyllable, the CV- prefixes /gɛ-/, /fa-/, /nɔ-/ were used with each cluster. The prefixes ensured a three-fold repetition of the same cluster type, for example, /gɛkl/, /fakl/, /nɔkl/ (see Orzechowska, 2019; Ulbrich et al., 2016; Wiese et al., 2017 for the use of the prefixes in similar studies). /g f n/ represent different consonant classes in terms of voicing, place, and manner features. Vowels /ɛ a ɔ/ are phonetically the most neutral as they represent symmetrical and maximally dispersed qualities in the low dimension of the vowel chart, where /ɛ ɔ/ are half-open and /a/ is open. In some stimuli, vowels were changed to avoid identity with real words: /gatr/ and /fɛwt/ were used to substitute for nominative/accusative singular *getr* /gɛtr/ (‘leggings’) and for genitive plural *fałd* /fawt/ (‘fold’). The final list included 198 stimuli; 15 types × 2 (EX-NEX) × 2 (WF-IF) × 3 (gɛ- fa- nɔ-), plus 3 plateaus × 2 (EX-NEX) × 3 (prefixes).

The stimuli were recorded by a phonetically-trained female, a native speaker of Polish. The recordings took place in a sound-proof cabin in the *Speech and Language Processing Laboratory* at the Faculty of English at AMU. The words were recorded with the MXL 770 microphone and the Roland Duo-Capture EX (24-bit digital) interface onto a Mac computer equipped with the waveform editor *Amadeus Pro* (2009) and were digitized at 44.1 kHz using a 16-bit resolution. To avoid untypical production of some articulatorily demanding NEX items, the recordings were evaluated by another linguist and re-recorded when necessary. The stimuli were cut using the *Praat* software (Boersma & Weenink, 2020).

### 4.6 Procedure

The task was for subjects to decide (as quickly as possible) whether a word heard sounded like a possible Polish word. Instructions read: “Decide if the word sounds as if it could exist in Polish.” Responses were provided by pressing either < left Ctrl > or < right arrow > . The assignment of the buttons to responses was randomized, that is < left Ctrl > and < right arrow > were “yes” and “no” buttons for subjects coded with even numbers, and “no” and “yes” for subjects coded with uneven numbers.

The stimuli were played to subjects via headphones. Reaction times were measured from the onset of the auditory stimulus to the response, with a timeout of 3,500 ms and an inter-trial interval of 2,000 ms. Missed trials were automatically coded as missing response, after which a new trial was initiated. The general timeline of a single trial is presented in Table 2.

**Table 2.** The Temporal Structure of a Single Trial.

Beginning of a trial (fixation star + blank screen)	Auditory stimulus + response	Transition between trials (blank screen)
= 1,300 ms	= 3,500 ms	= 2,000 ms

The stimuli were arranged in two blocks. It was ensured that three words featuring a specific CC type were split between the blocks. For example, /farɲ/ and /gɛps nɔps/ were assigned to block 1, while /gɛrɲ nɔrɲ/ and /faps/ were allocated to block 2. Randomizations were performed for cluster lists in each block and “yes”–“no” response button assignment. As a result, each subject was given a different version of the experiment. Before the experiment started, subjects were provided with a list of 10 training items to get familiar with the task.

The experimental session took place in the *Language and Communication Laboratory* (Faculty of English, AMU). Eight students were tested at a time. Subjects were monitored from a control room via a glass window and ceiling-mounted video cameras. When necessary, subjects were attended to by the experimenters during a break between the blocks. The total duration of the experiment was about 20 minutes. Reaction times and responses were registered by means of the *E-Prime* software (version 2.0, Schneider et al., 2002).

### 4.7 Phonetic analysis of stimuli

To exclude potential artifacts (resulting from the differences in the phonetic realization of words), we first compared the mean duration of the stimuli. Table 3 summarizes the results for each condition. For theoretical and methodological reasons, further discussed in Section 4.8, plateaus were merged with the class of IF clusters.

**Table 3.** Mean Duration of Stimuli (in s), Grouped by Condition.

Vowel	Existent		Non-existent	
	Well-formed	Ill-formed	Well-formed	Ill-formed
a	0.644	0.703	0.672	0.694
ɛ	0.590	0.674	0.647	0.654
ɔ	0.590	0.632	0.585	0.618

As the three-way ANOVA suggests, stimuli durations are significantly different across the sonority and vowel quality conditions, *F*(1, 186) = 41.838, *p* < .001 and *F*(2, 186) = 39.230, *p* < .001, respectively, and the sonority × existence interaction, *F*(1, 186) = 13.163, *p* < .001, while they do not differ significantly across existence, *F*(1, 186) = 2.149, *p* = .144, and other two-factor (sonority × vowel quality and existence × vowel quality) and three-factor interactions were also not significant, *F*(2, 186) < 0.573, *p* > .565 for each.

The largest differences are found between the WF-EX and IF-EX conditions, whereby EX-WF forms are shorter than EX-IF items. The extremes of duration range between 0.590 s for /ɔ/-EX-WF words and 0.703 s for /a/-EX-IF. As to vowels, the mean duration of /ɛ a ɔ/ words is significantly different in each condition. Although all Polish vowels are phonemically short, the phonetic variation can be explained by an inverse correlation between vowel height and intensity, such that lower vowels tend to be longer and more intense than higher vowels (Lehiste, 1970).

The phonetic differences between conditions are highly unlikely to affect subjects’ reactions to the clusters and the vowel because they are processed already at the beginning of the vowel, while the duration of the whole word is evaluated only at its end. Nevertheless, to ensure that the phonetic variation is not a confounding factor, the reaction time statistics in Section 4.9 (Tables 5 and 7) include stimulus duration as an independent variable. In addition, the influence of potential variation in other phonetic properties such as intensity and F0 is partly modeled by the inclusion of the “audio” random intercept in the random-effect structure. In consequence, the interpretation of the results makes it possible to abstract from the phonetic properties of vowels and focus on the phonological differences in height and frontness.

### 4.8 Methodological clarifications

The pre-processing of the data entailed eliminating trials coded as training and rest phase. From the total number of observations (*N* = 8,118), some were excluded: failure to react (*N* = 229) and reaction times lower than 400 ms (*N* = 103). The 400 ms cutoff point, established on the basis of visual inspection, eliminated obvious outliers and errors (e.g., reaction times at 6 ms). Responses and reaction times were based on all the remaining observations (*N* = 7,786), found in the range 418–2,996 ms. We considered both logarithmic transformation and Box-Cox transformation (with parameter of transformation equal to −0.5858586) of reaction times. The final model was based on the logarithmized values, as they displayed a near-normal distribution and improved model fit. In the analysis to follow, “RT” denotes the logarithmized reaction times, where higher RT corresponds to longer processing times.

Statistical analyses were conducted using the *R* software (v. 4.1.0) (R Core Team, 2021) and packages: *readxl* (Wickham & Bryan, 2019), *dplyr* (Wickham et al., 2021), *lme4* (Bates et al., 2015), *afex* (Singmann et al., 2021), *MASS* (Venables & Ripley, 2002).

For responses, logistic mixed-effects models (henceforth *GLME*s) were used. They were estimated using a function (f: *lme4:: glmer(family = binomial)*). *GLME*s were fitted by maximum likelihood method using Laplace approximation.

For reaction times, we ran a series of linear mixed-effects models (henceforth *LME*s) using a function (f: *lme4:: lmer*). *LME*s were fitted by restricted maximum likelihood (*REML*). *T*-tests of coefficients used the Satterthwaite’s method. The competing GLMEs and LMEs were compared in terms of the goodness-of-fit ratios and significance tests of parametric coefficients. The goodness of fit was determined using adjusted *REML*, deviance, and Akaike information criterion (henceforth AIC).

Based on a function (f: *afex:: allFit*), the *bobyqa* optimizer achieved the best performance and was used to construct both *GLME*s and *LME*s.

We did not exclude the possibility of statistical significance based on the premise that *p*-value exceeds .05. Although the .05 level is the ubiquitous threshold for statistical significance, our interpretation of the results rests upon the idea that the threshold is somewhat arbitrary, and that reporting, for instance, *p* = .08 as *potentially* significant is also methodologically valid (e.g., Wainer & Robinson, 2003; Wasserstein, 2016).

We ran models for two dependent variables: reaction times (a continuous variable expressed in ms, range = 418–2,996 ms, *M* = 1,278, *SD* = 410) and response (two levels: no vs. yes, coded as no = 0, yes = 1, referring to items which were regarded as unacceptable = “no” and acceptable = “yes” in Polish). The independent variables included: existence (two levels: NEX vs. EX; coded as NEX = 0 and EX = 1), sonority (two levels: IF vs. WF; coded as IF = 0, WF = 1), and vowel quality (three levels: /ɛ/ vs. /a/ vs. /ɔ/). Vowel quality was binarized (i.e., dummy variables were created), with /o/ serving as the baseline. For the sake of clarity, we constructed a technical ex/son variable, which takes the values of the four possible combinations of existence and sonority (four levels: NEX-IF vs. EX-IF vs. NEX-WF vs. EX-WF). The use of the ex/son variable has two advantages. First, it allows to effectively incorporate the interaction into the model. Second, the values of each group are expressed explicitly, eliminating the need to derive them from the interaction term. Thus, a technical ex/son variable with three coefficients (three groups and NEX-IF as a reference point) is included in the models, rather than existence, sonority, and existence × sonority separately. Note that, formally, this model is identical to the one with an explicitly declared interaction existence × sonority.

Moreover, the effect of stimulus duration was controlled by including the duration variable (a continuous variable expressed in s to avoid operating on distinctly different scales across the independent variables, which is strongly disfavoured in mixed-effects modeling; range = 0.4953–0.8827 s, *M* = 0.6405, *SD* = 0.0677). In RT models, we have also considered response as a control variable. However, this variable seemed to be insignificant and, as such, was excluded from the final model. By the “final model”, we mean the model that best explains the variables analyzed. That is, a full model was first estimated taking into account each of the variables mentioned (ex/son, vowel quality, duration; and response in the case of the model explaining RT). Next, the variables which turned out to be distinctly insignificant (*p*-value clearly exceeding .10) were excluded from the model in order to remove information noise and prevent overparameterization.

All the models also included appropriate random effects: intercepts for subjects (subject) and stimuli (audio) and slopes for subjects (except for the one for duration, whose inclusion significantly increased the correlations between random terms, possibly leading to the overparametrization of the model). Random slopes for stimuli were not included since each stimulus is always assigned the same values of the explanatory variables; making the inclusion of these slopes pointless.

As stated in Section 4.2, plateaus have been largely considered IF in phonological theory. Accordingly, only two sonority levels (i.e., WF vs. IF and plateaus) would be used in the analysis. In order to verify whether grouping IF and plateau clusters under a single class is empirically valid, the following models were compared: one with three levels of sonority (WF vs. IF vs. plateau), and two with two levels (where plateaus were classified either as WF or IF).

The decision to include plateaus in the IF category was fully consistent with the model for reaction times, and partly consistent with the model for response. There was no significant difference between two competing *LME*s: the one in which the plateau items were coded as IF and the one with 3 sonority levels—results of model comparison: *χ*^2^(17) = 13.12, *p* = .728; two-level model *AIC* = 337, three-level model *AIC* = 353), suggesting that the more complex model can be reduced to a model featuring only two sonority levels. At the same time, there were some grounds to assume that the model with 3 sonority levels is better fitted than the model with plateau CCs coded as WF, *χ*^2^(17) = 25.56, *p* = .083; two-level model *AIC* = 350. The results for response somewhat diverge from RTs, suggesting a slight advantage of the three-level model over plateau-to-IF-formalization, *χ*^2^(17) = 39.91, *p* = .001; two-level model *AIC* = 8,549, three-level model *AIC* = 8,543, and a drastic advantage over plateau-to-WF-formalization, *χ*^2^(17) = 121.38, *p* < .001; two-level model *AIC* = 8,630, three-level model *AIC* = 8,543. However, the *AIC* indicates only a slight advantage of the three-level model over the two-level model with plateaus assigned to IF. The two-level response model is much simpler, having a dozen fewer parameters (29 vs. 46). Therefore, it seems reasonable to choose this simpler model, which is only slightly inferior in terms of fit.

Hence, considering theoretical and methodological consistency, it is justified to select the IF coding of plateaus for both models.

### 4.9 Results

The presentation of the results starts with the subjects’ behavioral responses. The descriptive statistics in Table 4 show the averaged acceptability rates (= percent of pressing “yes”) per condition.

**Table 4.** Percentage of “Yes” Responses, Grouped by Conditions.

V	Existent			Non-existent		
	Well-formed	Ill-formed	Plateau	Well-formed	Ill-formed	Plateau
a	63%	38%	35%	50%	32%	22%
ɛ	65%	35%	41%	49%	32%	28%
ɔ	54%	32%	33%	41%	29%	29%

Subject responses suggest a bias toward “yes” in EX-WF clusters, while IF clusters are endorsed as potential words at the same rate disregarding their existence status. Note that acceptability ratings for EX-IF and NEX-IF forms are similar across different vowel qualities, with only a slight preference for EX. The rates for plateau clusters are highly similar to IF and highly different from WF, which corroborates their classification as IF. As to vowel quality, /ɔ/ is typically associated with the lowest acceptability rates.

To further explore the relationship between the variables, we ran statistical analyses on all responses to estimate the probability of considering an item as possible in Polish using *GLME*s including random intercepts and random slopes. The results are presented in Table 5. Characteristics of the random effects and selected measures of model quality are detailed in Appendix B.

**Table 5.** The Results of the Mixed-Effects Binomial Regression Model Explaining Responses (the GLME Model).

Term	Coefficient	Std. Error	*z* Statistic	*p*-value	Flag
(Intercept)	2.081	0.735	2.833	.005	**
EX/SON: EX-IF	0.440	0.175	2.512	.012	*
EX/SON: NEX-WF	0.876	0.228	3.837	< .001	***
EX/SON: EX-WF	1.410	0.276	5.114	< .001	***
Vowel: /a/	0.857	0.176	4.880	< .001	***
Vowel: /ɛ/	0.614	0.160	3.838	< .001	***
Duration	−5.787	1.126	−5.138	< .001	***

Significance flags: *** < .001; ** < .01; * < .05. Reference categories: /ɔ/ for vowel quality; NEX-IF for ex/son. The ex/son variable refers to existence/sonority groups: EX—existent; NEX—non-existent; WF—well-formed; IF—ill-formed.

In their phonological judgment, Polish speakers rely on the clusters’ sonority profile, existence status, and vowel type. The overall probability hierarchy capturing the acceptability of a form is the following: the most acceptable—EX-WF > NEX-WF > EX-IF > NEX-IF—the least acceptable. Each of the differences between the groups of the ex/son variable is significant: Table 5 includes comparisons against NEX-IF, which show an increasingly strong difference in coefficients (from 0.44 to 1.41) and higher significance for EX-IF (*z* = 2.512, *p* = .012), NEX-WF (*z* = 3.837, *p* < .001), and EX-WF (*z* = 5.114, *p* < .001), respectively. Comparisons with the remaining reference groups showing the significance of each difference (*z* > 2.138, *p* < .033 for each coefficient) are provided in Appendix C.

Speakers endorse items containing sonority-obeying clusters attested in Polish: WF and existent types substantially increase the probability for the subjects to accept a nonword as a possible one. In turn, IF and non-existent clusters significantly increase the likelihood of the subjects rejecting a nonce word as a potential Polish word. The scale suggests that, first, sonority-obeying clusters are largely favored, and, second, some facilitating influence is also exerted by the existence of a CC, although the effect is stronger for sonority: changing from IF to WF *ceteris paribus* changes the dependent variable by as much as ca. 0.9, while changing from NEX to EX only by ca. 0.4. In addition, note that there is no interaction between sonority and existence: the coefficient for each ex/son group can be explained with a high degree of accuracy using the coefficients for the individual variables alone (and a significance test in the same model, but built on the sonority × existence interaction instead of the ex/son variable also indicates the insignificance of the interaction: *z* = 0.380, *p* = .704).

Moreover, we found a similar effect of vowels /ɛ/ and /a/ versus /ɔ/. The presence of /ɛ a/ increases the probability of judging a form as possible compared to /ɔ/. This probability is slightly higher for /a/ than /ɛ/ in the sample, but in this study there is too weak a basis for considering the difference to be significant (results of *Z* test of the /a/ coefficient in the model with /ɛ/ as a reference category: *z* = 1.550, *p* = .121). This leads to the following hierarchy of preferability: the most acceptable—/a/ ⩾ /ɛ/ > /ɔ/—the least acceptable. This result suggests that vowel /ɔ/ hinders the “yes” response in the decision-making process. The findings are consistent with the data in Table 4.

Apart from collecting responses, we measured reaction times. Let us start with an overview of the descriptive statistics, presented as Table 6. In the sample, EX-WF clusters are processed the shortest (*M* = 1,247 ms), while NEX-WF are processed the longest (*M* = 1,312 ms). Among vowels, words containing /ɔ/ evoke the shortest response latencies, containing /a/—the longest, and /ɛ/—merging between the two classes. This pattern holds for all the conditions with the exception of EX-WF. The overall mean (for the whole sample) of reaction times is *M* = 1,241 ms for /ɔ/, *M* = 1,270 ms for /ɛ/, and *M* = 1,325 ms for /a/.

**Table 6.** Mean Raw Reaction Times (in ms), Grouped by Conditions.

V	Existent				Non-existent			
	Well-formed	Ill-formed	Plateau	Total	Well-formed	Ill-formed	Plateau	Total
a	**1,282**	1,357	1,298	1,319	1,351	**1,317**	1,289	1,330
ɛ	**1,223**	1,287	1,219	1,250	1,322	**1,261**	1,266	1,290
ɔ	**1,239**	1,254	1,210	1,243	1,261	**1,221**	1,216	1,238
Total	**1,247**	1,300	1,243	1,269	1,312	**1,266**	1,257	1,278

Values given in bold represent the values that are most similar to the Plateau group.

Raw reaction time data are ambiguous in terms of plateaus’ affinity to WF or IF CCs. In Table 6, values given in bold type indicate the categories’ similarity to plateaus. The pattern is consistent for each existence class: NEX plateaus are processed more similarly to IF CCs, while EX plateaus are processed more similarly to WF CCs. However, this dissimilarity does not undermine the conclusions drawn from comparing the three-level and two-level models, as indicated earlier.

The LME model for RT is summarized in Table 7. Tests of significance give strong premises to infer the significance of the difference between the NEX-WF group, compared to other groups, *t*(66.8) = 3.213, *p* = .002. In contrast, no significant differences are observed for comparisons of the EX-WF versus NEX-IF, *t*(52.0) = 0.083, *p* = .934, and EX-IF versus NEX-IF, *t*(60.8) = 1.334, *p* = .187. More specifically:

(1) EX-WF and NEX-IF clusters entail the shortest reaction times for subjects to respond to. That is, it is the easiest for Polish speakers to judge the possibility of EX-WF and NEX-IF clusters occurring in Polish compared to other cluster types.
(2) NEX-WF clusters entail the longest reaction times for subjects to answer to. That is, it is the most cognitively challenging for Polish speakers to judge the existence of NEX-WF.
(3) The present study gives no indication of an EX-IF difference relative to the two groups with the shortest response time, *t*(60.8) = 1.334, *p* = .187. At the same time, both the *p*-value and the coefficient (0.013) for this group appear to be different from EX-WF, which may suggest that there is some basis for recognizing the relationship in future studies, but which cannot be detected with the sample tested here.
(4) The overall scale of reaction times is the following:
  the longest—NEX-WF > EX-IF = EX-WF = NEX-IF—the shortest.^4^

**Table 7.** The Results of the Mixed-Effects Linear Regression Model Explaining Logarithmized Reaction Times (the LME Model).

Term	Coefficient	Std. Error	*t* Statistic	df	*p*-value	Flag
(Intercept)	6.909	0.048	144.900	193.549	<.001	***
EX/SON: EX-IF	0.013	0.010	1.334	60.836	.187	
EX/SON: NEX-WF	0.038	0.012	3.213	66.800	.002	**
EX/SON: EX-WF	0.001	0.017	0.083	51.990	.934	
Vowel: /a/	0.050	0.010	4.965	98.601	<.001	***
Vowel: /ɛ/	0.016	0.009	1.865	87.201	.065	○
Duration	0.265	0.063	4.171	191.197	<.001	***

Significance flags: *** < .001; ** < .01; ○ < .1. Reference categories: /ɔ/ for vowel quality; NEX-IF for ex/son. The ex/son variable refers to existence/sonority groups: EX—existent; NEX—non-existent; WF—well-formed; IF—ill-formed.

As for vowels, we found significant differences between /a/ and /ɔ/, *t*(98.6) = 4.965, *p* < .001, and some evidence for the difference between /ɛ/ and /ɔ/, *t*(87.2) = 1.865, *p* = .065. The difference between /a/ and /ɛ/ is also significant, *t*(191.2) = 3.921, *p* < .001. The presence of /a/ evokes longer response latencies than /ɔ/, and /ɛ/ has a comparable effect, albeit much weaker (0.05 vs. 0.016 change in RT).

As has been mentioned before, when testing models, we used response as a control variable, but it was not included in the final model for reaction times. The effect of response was non-significant in the model including it and the corresponding random slopes, *t*(39.7) = 1.44, *p* = .157. Thus, the variable was removed from the final model. The results are compatible with those in Table 6. For full characteristics of random effects and the fit indices of the model, see Appendix B.

## 5 Discussion

The goal of the paper was to report on a reaction time study on word-final CC clusters and neighboring vowels in Polish. We tested how cluster existence, cluster sonority, and vowel quality influence the likelihood of Polish native speakers endorsing a nonce word as being a potential Polish word. On the one hand, the findings confirm the previous results that existence and well-formedness facilitate online processing (Section 5.1). On the other hand, they testify to the critical role of vowel quality in the speed of lexical access (Section 5.2). Section 5.3 discusses the results in relation to other studies on Polish. The ramification of the findings is outlined in Section 5.4, where an alternative explanation of the cognitive behavior of speakers is presented.

### 5.1 Existence and sonority

First of all, EX-WF, NEX-IF, and EX-IF clusters involve shorter response latencies, revealing that they are cognitively less costly to recognize and evaluate, while NEX-WF clusters elicit longer response latencies, suggesting greater processing effort for the decision-making process. Overall, ill-formedness contributes to rapid evaluation of clusters, while the difficulty of evaluating WF clusters depends on their (non-)existence status. Acceptability rates partially mirror the pattern: words with EX-WF clusters are largely considered to be legitimate Polish words, making them cognitively more accessible. Forms which tend to be rejected as Polish words contain sonority-offending CCs; thus sonority violations constitute the prime benchmark in cluster evaluation, rendering these forms easy to judge. Moreover, the fact that NEX-WF items are most difficult to judge suggests that the sonority profile is an important cue in phonological decision. Thus, the non-existent status of WF clusters leads to cognitive dissonance during processing. This result is compelling; it might reflect the clusters’ markedness status in the cognitive grammar of native speakers of Polish.

It must be borne in mind that the relationship between existence and sonority is critical for the understanding of the cognitive representation of clusters. In the present case, the contribution of each of the variables considered separately is insufficient to account for the holistic relations regulating the active decision-making process. This observation is based on parametric coefficients in Table 7 in Section 4.9, where an unexpected sonority rise influences reaction times only in the class of non-existent clusters. Note that this result can be compared to the early time window (N400) in session 1 reported in an ERP study in Wiese et al. (2017, see left panel in Figure 4 of that study, p. 10). Electrophysiological reactions in this time window can reflect phonological discrimination, which is explicitly investigated in the present study. Although Wiese et al. (2017) do not report significant differences for the existence × sonority interaction, the descriptive statistics in Figure 4 display the same pattern: for the WF group, NEX clusters entail the greatest cognitive effort. In this context, our study can be seen as a further exploration of this relationship, making it possible to determine the significance of the differences that were observed in Wiese et al. (2017) only at the level of descriptive statistics.

Moreover, the present study offers insights into the processing of plateau clusters. Although clusters lacking sonority distance are generally deemed marked—since a minimum sonority difference between two adjacent positions in a syllable is required (e.g., Clements, 1990; Parker, 2002; Steriade, 1982)—the absence of sonority distance was argued to be universally unproblematic in Berent et al. (2007). Also in Polish, a lack of minimal sonority distance was permitted in obstruent onsets (with the exception of geminates) in Rubach and Booij (1990). This constraint reflects a syllabification algorithm, according to which a plateau such as /tk/ in *matka* “mother” tends to be tautosyllabified (*ma.tka*) rather than heterosyllabified (*mat.ka*) by native speakers. Further theoretically-based exclusions of plateaus were proposed in Rochoń (2000), who posited constraints prohibiting the occurrence of onsets featuring two nasals, liquids or glides. The statistical modeling in the present paper lends support to Government Phonology and Optimality Theory constraints by showing that reactions to plateaus largely parallel reactions to sonority-violating clusters. This finding shows that at least a minimal sonority distance is required in word-final phonotactics in Polish, further supporting principles mandating a minimal sonority distance (Harris, 1983; Selkirk, 1984; Steriade, 1982). Stating whether this behavior is systematic and symmetrical requires further research focusing on the role of distances in Polish onsets and codas, which we are currently pursuing.

### 5.2 Vowel quality

A novel finding is that vowel quality is a significant predictor of the speed of cognitive access in Polish speakers, with /ɔ/ displaying the strongest facilitative effect on processing, in opposition to /a/. The hierarchy established for “yes” responses is shown in (2), where the probability of accepting a form as a potential Polish word was facilitated more by /a ɛ/ than by /ɔ/. These findings can be integrated into the theory on sonority distinctions between vowels and theoretical approaches to phonotactics, which we discuss in the following sections.

The results suggest that the discussion of phonotactics can well incorporate the phonological characteristics of vowels, although very few contributions have used both vowel quality and cluster well-formedness in the analysis of syllable structure (e.g., Parker, 2008). Typically, studies on phonotactics have used vowels as a benchmark for the evaluation of cluster formedness (preceeding vowels or following them), without considering their sub-segmental properties. The phonotactic principle that takes into account vowel quality in the computation of cluster preferability is *Net Auditory Distance* (henceforth NAD, e.g., Dziubalska-Kołaczyk, 2019; Dziubalska-Kołaczyk et al., 2014), in which a distinction is made between vowels of different height and frontness. This model predicts that a structure such as /ɛ/CC is more preferred than /a/CC or /ɔ/CC in Polish, disregarding a cluster type. This prediction partially aligns with the results for the response variable, whereby monosyllables containing vowel /ɛ/ were shown to be more acceptable as Polish words than monosyllables containing /ɔ/. Further differences between the present results and NAD can be attributed to different feature preferences that underlie lexical, perceptual, cognitive, and articulatory phonotactics, as argued in Orzechowska (2019).

Previous research has highlighted the word-final or coda position as particularly important for understanding the relationship between vowels and consonants. For example, in CVC strings in English, vowels were shown to display greater cohesion with the following consonant, especially if this consonant was represented by a liquid (e.g., Stemberger, 1983; Treiman, 1984). Experimental work of Treiman (1984) revealed that native speakers tend to group the postvocalic liquid with the vowel, while consonant clusters beginning with obstruents form cohesive units on their own. These results support the psychological reality of the sonority hierarchy, which we have also reported in the current study on Polish.

Moreover, the observed latencies and behavioral responses to vowel quality tip the balance in favor of sonority scales, which capture a degree of granularity within the class of vowels (for an overview see Parker, 2017). The universal sonority scale generally predicts an increase in loudness from high to low for peripheral vowel qualities. In the present analysis, two vowel hierarchies emerge based on the reaction time and response data, as summarized in (2).

(2) Preferred vowel hierarchies
 (a) the cognitive domain: the greatest effort—/a/ > /ɛ/ > /ɔ/—the least effort
 (b) the metalinguistic domain: the most probable—/a/ ⩾ /ɛ/ > /ɔ/—the least probable

As can be seen in (2a), half-open back /ɔ/ facilitates processing the most, followed by half-open front /ɛ/, while the most sonorous open low /a/ leads to slower access times. In contrast, the most cognitively costly vowel /a/ is favored by Polish speakers at the metalinguistic level (2b). Moreover, in (2b), the preferability of /a/ and /ɛ/ is comparable. The rejection of nonce words featuring /ɔ/ might suggest that the front-back distinction (rather than the universally expected high-low distinction) is consulted in the decision-making process. We can, thus, conclude that Polish speakers use fine-grained distinctions mainly in frontness (i.e., front /ɛ/ and central /a/ vs. back /ɔ/), rather than in height (i.e., lower /a/ vs. higher /ɛ ɔ/), in which /ɛ/ and /ɔ/ form a joint group. This result is consistent with a recent study on German *qu*-words (Orzechowska & Wiese, 2024). The processing of the initial phonemic cluster, phonetically realized as either [kf- kv- kʋ-], is facilitated by vowels based on their front-back relation, rather than on the widely acknowledged height dimension.

Finally, it must be noted that the findings of the study trigger questions on the representation of syllable structure in the mental lexicon of Polish speakers. For English, studies based on polysyllables (Fowler et al., 1993; Treiman et al., 1995) provided compelling evidence supporting the hierarchical organization of the syllable, in which the rhyme consists of a vowel and the remaining consonantal material. Although the results of the present study testify to the psychological reality of the sonority principle and degrees of vowel frontness, they cannot be generalized to the rhyme unit. As Davis (1989) rightly argued, conclusions drawn on the structure of monosyllables may be well interpreted as effects characteristic of the word. This means that we are not able to unambiguously interpret the present findings in relation to the onset/rhyme division of the syllable. Moreover, the stimuli were controlled for the phonological properties of vowels and clusters in isolation, but not for rhymes. For this reason, we refrain from making claims on the internal organization of the syllable in Polish, and declare it as a limitation of the study. This question remains open for further research.

### 5.3 Conflicting results on sonority—an interpretation

Placing the present study in a broader empirical context shows that in spite of the fact that native speakers of Polish are continuously exposed to sonority-offending clusters (see examples in (1)), the effects of sonority are reserved for selected cognitive functions. Based on the literature overview and her empirical work, Orzechowska (2019) suggested that although the sonority principle is available to Polish speakers, it is restricted to specific experimental tasks, for instance, by being applicable in learning and memorization, but not in phonological discrimination. Let us outline some competing evidence from syllabification and production studies.

Syllabification in Polish was argued to be sonority-driven in, *inter alia*, Bethin (1992), Cyran and Gussmann (1999) and Cetnarowska and Żygis (2007). However, a number of studies showed that sonority-based syllabification is far too simple. Szpyra-Kozłowska (1998) reported a high degree of variability in phonetic division of medial clusters, and Szpyra-Kozłowska (2000) observed that other factors (such as morphology) might be involved. In addition, the effects of sonority seem to be constrained by prosodic position. While sonority was shown to play a crucial role in the phonetic syllabification of prefixed words in Cetnarowska and Żygis (2007), Szpyra-Kozłowska (2000) argued that Polish speakers also consider highly productive and fully transparent suffixes in their parsing strategies. Similarly in production studies, sonority was shown to affect acquisition of clusters (e.g., Łukaszewicz, 2010; Jarosz, 2017), while other factors were shown to affect spontaneous speech in adults. For instance, phonostylistic processes in adults tend to be modulated by cluster frequency in word initial position, and features such as stridency, coronality, or vowel quality in word-final position (Orzechowska, 2019). Furthermore, the psycholinguistic studies discussed in Section 3 point to a division of labor at the neuro-cognitive level, whereby sonority facilitates learning and recollection, but is inactive in phonological discrimination (Wiese et al., 2017). This result suggests that other principles might be active when Polish speakers engage in phonological judgment.

Overall, the body of accumulated evidence indicates that the sonority principle is generally available to Polish speakers; however, its retrieval depends on a type of a linguistic modality” activated. The role of the sonority principle in Polish is task-dependent and should be interpreted only in relation to a specific information-processing task. When viewed from this perspective, the findings of the current study have wide-ranging implications for phonological research and phonotactic models, to be discussed in Section 5.4.

Another potential explanation is that the effect of the sonority slope depends on the control of other variables in statistical models. Thus, it is strongly advisable that future research includes hierarchical models, which make it possible to trace the effects of additional variables on the processing of clusters. Sonority distances are one of such variables. In principle, clusters displaying a large sonority distance are easier to recognize and process than clusters characterized by a small distance (e.g., Berent et al., 2014; Moreton, 2002; Pitt, 1998; for further references, see Section 3). Another factor that was shown to affect the processing of phonotactics is the morphological status of clusters, whereby morphologically complex clusters facilitate processing and are prone to fewer errors (e.g., Korecky-Kroll et al., 2014).

### 5.4 Implications for phonological theory

Highly significant parametric coefficients of the existence variable can be interpreted in two ways. On the one hand, the results can be taken as indirect evidence in favor of probabilistic factors in the study of phonotactics, where emphasis is placed on frequency and segment co-occurrence (both vowels and consonants). Under this view, Polish speakers should be sensitive to probabilistic frequencies of substrings of clusters and their prosodic position. This type of relationship is accounted for in lexical models on phonotactics, which measure likelihood co-occurrence of substrings of words based on neighborhood density, n-grams, and syllable parsers (e.g., Bailey & Hahn, 2001; Vitevitch & Luce, 1999). The studies have demonstrated that the frequency with which a segment occurs in a given syllable or word position, and co-occurs with other segments, is stored in the memory and is correlated with accuracy of judgment and response latencies.

Work on segmental neighbors revealed that spoken words are easier to recognize when phonologically similar words are activated in memory. Speakers provide delayed and less accurate responses to real items in high-frequency and/or dense neighborhoods, compared to words that occur in lower frequency and/or lower density phonological neighbors. The reverse pattern is found for nonce words (Luce & Pisoni, 1998). Although the data analyzed in this paper do not make it possible to address this question for Polish consistently, some comparison can be made. All nonce monosyllables presented to speakers have frequency equal to zero, while stimuli containing existent CCs naturally offer greater lexical support. As a consequence, we should expect a statistically significant difference in the processing of EX and NEX targets. In line with Luce and Pisoni (1998), monosyllables ending in existent clusters should be processed faster. As shown in Section 4.9, a difference between EX and NEX items is attested in relation to well-formedness, where EX-WF and NEX-IF involve the shortest reaction times. Stimuli featuring EX clusters display greater inter-segmental frequency than NEX clusters, which results in an increase in the activation level of the acoustic patterns heard. In turn, this activation leads to shorter response latencies.

The observed sensitivity to vowel characteristics shows that the evaluation of nonce words involves multiple levels: Apart from the phonological structure of final clusters, Polish speakers consult feature-based grammaticality. This interpretation has received great support from previous research, which stressed the relevance of sub-segmental cues in computing phonological information. Numerous approaches proposed to describe language structure using abstract feature patterns to describe and predict phonological structures and model the learning paths of language users. For instance, Hayes and Wilson (2008) proposed a phonotactic learner that operates on constraints constructed on the basis of natural classes and showed that it can successfully acquire the grammar of constraint-based languages based on several phonological features. Albright and Hayes (2003) argued that phonotactic grammar is discovered by learners in the process of identifying abstract feature patterns, which are shared over pairs of strings. The idea was also captured in Albright (2009) who proposed a feature-based approach to gradient phonological judgments, whereby the acceptability of nonce forms is based on the most probable combination of natural classes.

Given the present data, we can conclude that phonotactic intuitions, at least at some level of processing, rely on feature-based phonological representations. Apart from vowel features, well-formedness is also expressed in terms of manner features. The sonority scale, disregarding the degree of detail that it involves, embraces undisputed junctures between vowels and sonorants as well as between sonorants and obstruents captured by features [± consonant], [±sonorant]. Finer, and language-specific differences are related to the classification of rhotics and laterals, affricates and stops/fricatives, and the voiced and voiceless contrast. Thus, we argue that cognitive representations cannot be discussed in isolation from the sub-segmental structure of sounds and their sequences, and that cognitive processes operate on high-level constraints.

The feature-based interpretation questions a body of research that advocates for segmental representations, and distinctiveness of vowels and consonants. It has been shown that the processing of vowels and consonants (or clusters) requires access to different processing levels or mechanisms, and that vowels and consonants are represented independently in the mind of speakers. Evidence in favor of this interpretation was offered by Caramazza et al.’s (2000) study on aphasic speakers, which led to the conclusion that vowels and consonants are categorically distinct entities at the underlying level, in spite of their phonetic similarities. Roelofs’s (1999) articulatory data also revealed that segments, rather than features, facilitate word planning, which suggests that phonological encoding involves vowels and consonants as distinct entities. A neurolinguistic study by Boatman et al. (1997) corroborated the hypothesis: At the cortical level, consonant and vowel perception represent distinct perceptual phenomena. Further evidence in favor of the distinction was provided in studies on phonotactic learning. Newport and Aslin (2004) demonstrated that listeners easily acquire patterns between non-adjacent consonants, which required skipping over vowels. Similarly, Chambers et al. (2010) showed that learners rapidly recognize CVC generalizations disregarding the vowel quality. Overall, the results support the distinction between vowels and consonants as basic functional units (see Nespor et al., 2003 for a detailed discussion). At present, the verification of this proposal is not possible as it would require another study designed specifically to address the dissociation between vowels and consonants. It might be the case, however, that depending on a task, Polish speakers access different phonological levels in their decision units.

It must be stressed that the present conclusions hold for the final, and thus prosodically weaker, word position. Extensions of this study will involve investigating native speakers’ sensitivity to the sub-constituents of the syllable in polysyllabic words, as well as their sensitivity to vowels and gradient markedness in terms of sonority distances in both word positions. Further work on initial phonotactics has the potential of revealing whether the cognitive behavior of Polish speakers is symmetrical. As to statistics, apart from the aforementioned hierarchical regression, future analyses could involve quantile regression as it offers an in-depth analysis of predictors by looking into consecutive fragments of the distribution of the observed latencies.

## 6 Conclusions

The goal of the paper was to investigate psychological factors that contribute to the processing of final CC clusters in Polish. The literature on the topic is sparse and has mainly focused on cluster existence and well-formedness. We expanded upon previous analyses by testing whether phonotactic intuitions are affected not only by cluster status and its positive or negative sonority slope, but also by the quality of the vowel preceding the sequence. The results of the analysis are twofold. First, we demonstrated that universally unmarked syllables in terms of sonority are easily identified as possible and involve fewer errors, while marked syllables are easily identified as unlikely to occur in Polish. The fact that speakers experienced difficulties judging sonority-violating clusters as possible suggests that the sonority principle is part of speakers’ phonological knowledge. Second, we observed a systematic perceptual contribution of vowels to the evaluation of novel forms. The paper also offers evidence in favor of the fact that phonological information and phonological access in a phonotactically complex language require feature representations, and that cognitive processes operate on high-level phonological information.

## Appendix

**Appendix A:** A List of Phonetically-Transcribed CVCC Nonce Words Used in the Study.

Monosyllables with existent clusters					
Well-formed			Ill-formed		
gɛ-	fa-	nɔ-	gɛ-	fa-	nɔ-
gɛrɲ	farɲ	nɔrɲ	gɛnr	fanr	nɔnr
gɛɲɲ	faɲɲ	nɔɲɲ	gɛsm	fasm	nɔsm
gɛfɲ	fafɲ	nɔfɲ	gɛfn	fafn	nɔfn
gɛft	faft	nɔft	gɛtf	fatf	nɔtf
gɛsp	fasp	nɔsp	gɛps	faps	nɔps
gɛsk	fask	nɔsk	gɛkx	fakx	nɔkx
gɛjs	fajs	nɔjs	gɛsw	fasw	nɔsw
gɛŋk	faŋk	nɔŋk	gɛpɲ	fapɲ	nɔpɲ
gɛrʦ	farɲ	nɔrɲ	gɛtm	fatm	nɔtm
gɛlk	falk	nɔlk	gɛkl	fakl	nɔkl
gɛrk	fark	nɔrk	gatr	fatr	nɔtr
gɛwɲ	fawɲ	nɔwɲ	gɛpl	fapl	nɔpl
gɛwp	fawp	nɔwp	gɛpw	fapw	nɔpw
gɛwt	fawt	nɔwt	gɛtw	fatw	nɔtw
gɛwk	fawk	nɔwk	gɛkw	fakw	nɔkw
Monosyllables with non-existent clusters					
Well-formed			Ill-formed		
gɛ-	fa-	nɔ-	gɛ-	fa-	nɔ-
gɛln	faln	nɔln	gɛmr	famr	nɔmr
gɛxʨ	faxʨ	nɔxʃ	gɛʦf	faʦf	nɔʦf
gɛsts	fasts	nɔsts	gɛʨs	faʨs	nʨs
gɛfp	fafp	nɔfp	gɛpf	fapf	nɔpf
gɛxp	faxp	nɔxp	gɛpx	fapx	nɔpx
gɛʃk	faʃk	nɔʃk	gɛtx	fatx	nɔtx
gɛjz	fajz	nɔjz	gɛzj	fazj	nɔzj
gɛmg	famg	nɔmg	gɛbn	fabn	nɔbn
gɛrʤ	farʤ	nɔrʤ	gɛgn	fagn	nɔgn
gɛlg	falg	nɔlg	gɛgl	fagl	nɔgl
gɛrg	farg	nɔrg	gɛʥr	faʥr	nɔʥr
gɛwʣ	fawʣ	nɔwʣ	gɛʣw	faʣw	nɔʣw
gɛjb	fajb	nɔjb	gɛbj	fabj	nɔbj
gɛjd	fajd	nɔjd	gɛdj	fadj	nɔdj
gɛjg	fajg	nɔjg	gɛgj	fagj	nɔgj

Rows represent matching WF-IF cluster pairs for each existence condition.

**Appendix B:** The Random-Effects Statistics and Fit Indices for the Model Explaining the response Variable *(GLME)*.

Groups	Term	*SD*	Correlation matrix				
Audio	(Intercept)	0.713	(Intercept)	EX-IF	NEX-WF	EX-WF	Vowel/a/
Subject	(Intercept)	1.387					
	EX/SON: EX-IF	0.426	−.648				
	EX/SON: NEX-WF	0.982	−.520	.738			
	EX/SON: EX-WF	1.325	−.579	.784	.960		
	Vowel: /a/	0.287	−.625	.791	.536	.736	
	Vowel: /ɛ/	0.330	−.259	.843	.467	.431	.434
Residual		0.241	Min	1Q	Median	3Q	Max
Scaled residuals			−5.071	−0.633	−0.284	0.699	6.253
Model		AIC	BIC	LogLik	Deviance		
		8548.5	8750.4	−4245.3	8490.5		

The Random-Effects Statistics and Fit Indices for the Model Explaining the RT Variable *(LME)*.							
Groups	Term	*SD*	Correlation matrix				
Audio	(Intercept)	0.025	(Intercept)	EX-IF	NEX-WF	EX-WF	Vowel/a/
Subject	(Intercept)	0.169					
	EX/SON: EX-IF	0.026	−.364				
	EX/SON: NEX-WF	0.047	−.073	.675			
	EX/SON: EX-WF	0.089	−.216	.762	.986		
	Vowel: /a/	0.022	−.750	.284	−.076	.072	
	Vowel: /ɛ/	0.015	−.191	.133	−.184	−.092	.790
Scaled residuals			Min	1Q	Median	3Q	Max
			−4.327	−0.651	−0.104	0.552	4.744
Model REML criterion		324.3	AIC	BIC	LogLik	Deviance	
Model after refitting using ML			337.33	546.14	−138.67	277.33	

**Appendix C:** The Results of the Comparisons of ex/son Groups in the response Model *(GLME)* Against Different Reference Groups.

Reference Group	Group	Coefficient	Std. Error	*z* Statistic	*p*-value	Flag
EX-IF	NEX-IF	−0.440	0.175	−2.512	.012	*
	NEX-WF	0.436	0.204	2.139	.032	*
	EX-WF	0.971	0.246	3.945	< .001	***
NEX-WF	EX-IF	−0.436	0.204	−2.139	.032	*
	NEX-IF	−0.876	0.228	−3.835	< .001	***
	EX-WF	0.535	0.192	2.786	.005	**
EX-WF	NEX-IF	−1.410	0.276	−5.110	< .001	***
	EX-IF	−0.971	0.246	−3.946	< .001	***
	NEX-WF	−0.535	0.192	−2.786	.005	**

Significance flags: *** < .001; ** < .01; * < .05.

## Notes

1. This study was conducted in parallel to an experiment on initial phonotactics, in which high front vowels /i ɨ/ were eliminated to avoid their palatalizing effect on the preceding C. Since the two experiments are to be compared, high vowels were not considered as nuclei of stimuli at all.

2. Log freq = 6 encompasses the most-frequent clusters, while log freq = 1 embraces clusters with few tokens.

3. Since the rule of final obstruent devoicing applies in Polish, clusters ending in voiced plosives, fricatives, and affricates were regarded as NEX.

4. As stated in (3), the basis to assume the equality of EX-IF and EX-WF is weaker than that of EX-WF and NEX-IF.
